# Investigating the impact of STING pathway activation on breast cancer treatment outcomes: development and validation of a prognostic model

**DOI:** 10.3389/fimmu.2024.1438364

**Published:** 2024-08-09

**Authors:** YangYan Zhong, Hong Cao, Wei Li, Jian Deng, Dan Li, JunJie Deng

**Affiliations:** ^1^ The Second Affiliated Hospital, Department of Breast and Thyroid Surgery, Hengyang Medical School, University of South China, Hengyang, Hunan, China; ^2^ Clinical Research Center for Breast and Thyroid Disease Prevention and Control in Hunan Province, Hengyang Medical School, University of South China, Hengyang, Hunan, China

**Keywords:** breast cancer, STING, tumor microenvironment, single-cell analysis, prognosis

## Abstract

**Introduction:**

Breast cancer (BRCA) is a significant cause of cancer-associated mortality across the globe. Current therapeutic approaches face challenges such as drug resistance and metastasis. Immune signaling is triggered by chromosomal instability (CIN) generates misplaced DNA structures that activate the cyclic GMP–AMP synthase–stimulator of interferon genes (cGAS-STING) pathway, triggering. Studies have linked STING activation to BRCA treatment.

**Methods:**

The bulk RNA-seq data for patients with BRCA were collected from the TCGA-BRCA cohort, GSE20685, and GSE96058 cohorts. STING pathway-related genes (SRGs) were obtained from the Reactome database. Differentially expressed genes were analyzed using the limma package. Immune cell infiltration was analyzed using the IOBR package. Gene Ontology biological processes, Kyoto Encyclopedia of Genes and Genomes pathways, and cancer hallmark pathways were analyzed using the MSigDB database. Prognostic models were prepared using the least absolute shrinkage and selection operator and multiple-factor Cox regression analysis. Single-cell analysis was performed using the Seurat and SCP pipeline.

**Results:**

The expression patterns and clinical relevance of SRGs were analyzed in patients with BRCA. Transcriptional differences in the SRGs were observed between normal and tumorous tissues, with global down-regulated STING1 and up-regulated TBK1 in BRCA tissue. Tumor tissues were classified through consensus clustering analysis into two distinct groups, with differences in clinical characteristics and immune infiltration. A prognostic model related to the differences in STING pathway activity—high prognostic stratification potency—was developed and validated. Correlation analysis revealed suppressed overall immune activation in patients with BRCA having higher risk scores. Gemcitabine had a more favorable outcome in the low-risk group. The activity of the prognostic model at the single-cell level was confirmed through single-cell analysis, particularly in CD8 T cells and intratumor natural killer cells.

**Conclusion:**

A STING pathway-related prognostic model developed and validated and the model could accurately predict BRCA patient outcomes. These findings have important implications for the personalized treatment and management of patients with BRCA.

## 1 Introduction

The incidence of breast cancer (BRCA) has reached an approximately estimated mortality of 0.7 million across the globe (1). While several methods, such as mammectomy and chemoradiotherapy drugs, have been developed for the treatment and management of BRCA, there are still challenges to overcome. One such challenge is drug resistance, which restricts the ability of chemotherapy agents like doxorubicin (DOX) in suppressing BC progression (2). Besides drug resistance, metastasis is a major factor contributing to the poor prognosis of patients with BRCA (3). Understanding the inherent mechanisms that trigger BRCA development is crucial for developing effective treatments.

One key feature of cancer is chromosomal instability (CIN), which leads to the generation of misplaced DNA structures called micronuclei and chromatin bridges. These structures activate the cyclic GMP–AMP synthase–stimulator of interferon genes (cGAS-STING) pathway, triggering immune signaling and potentially eliminating cancer cells (4). However, cancers with high CIN often evade immune responses and exhibit metastatic behavior and poor outcomes. The cGAS-STING signaling pathway has attracted significant attention in cancer immunotherapy. In BRCA treatment, paclitaxel has been linked to the activation of the cGAS-STING pathway, implying that targeting this pathway may be a potentially novel therapeutic strategy (5, 6). Overall, understanding and manipulating the cGAS-STING pathway holds promise for improving BRCA treatment.

In this study, we developed a prognostic model based on the STING pathway-related dysregulated genes. We used the least absolute shrinkage and selection operator (LASSO) and multiple-factor regression analysis to select the genes of the most prognostic significance for the model construction. The model exhibited high prognostic stratification potential in the training set and was validated in two external datasets. In the high-risk group, enhanced expression of various STING pathway genes was observed. We found the association of higher risk scores with suppressed immune activation and altered immune cell infiltration. At single-cell resolution, a highly activated signature related to the STING pathway was identified in the CD8 T cells and intratumor NK cells. Our study provides extensive insights into the transcriptional changes, molecular subtypes, dysregulated pathways, and clinical implications of STING-pathway-related genes in BRCA.

## 2 Methods

### 2.1 Data origin

The bulk RNA-seq data for patients with BRCA were collected from The Cancer Genome Atlas Breast Invasive Carcinoma (TCGA-BRCA) cohort using the TCGAbiolinks R package, GSE20685 and GSE96058 cohorts using the GEOquery R package. These datasets contain single-cell RNA-seq (scRNA-seq) data for BRCA. We obtained single-cell RNA-seq (scRNA-seq) data for BRCA from three datasets from the Gene Expression Omnibus (GEO) database (https://www.ncbi.nlm.nih.gov/geo/). These datasets were chosen to comprehensively analyze gene expression patterns in BRCA across different research studies and omics.

### 2.2 Acquisition of STING-pathway-related gene sets

The STING-pathway-related genes (SRGs) were acquired from the R-HSA-3134800 of the Reactome database (https://curator.reactome.org/) to evaluate their effect on the STING pathway activity.

### 2.3 Differential gene analysis

Differential gene analysis was performed using the limma package with a significance threshold of adj.P.val. < 0.01 and fold change (|logFC|) > 1.

### 2.4 Analysis of immune cell infiltration

For this, the IOBR R package (https://github.com/IOBR/IOBR) was utilized. We employed the built-in tumor microenvironment (TME) analysis algorithms, namely CIBERSORT and ESITMATE, to examine the bulk RNA-seq dataset.

### 2.5 Identification of immune checkpoint genes (ICGs) and chemotherapy sensitivity

The immune checkpoint genes and chemotherapy sensitivity-related gene sets were retrieved from the literature (7).

### 2.6 Analysis of gene ontology-biological processes (GO-BP), Kyoto Encyclopedia of Genes and Genomes (KEGG), and cancer hallmarks

The MSigDB database (https://www.gsea-msigdb.org/gsea/msigdb/) was utilized for the analysis of GO-BP, KEGG pathways, and cancer hallmark pathways.

### 2.7 Mutation analysis and prediction of drug sensitivity

The mutation was analyzed using the maftool R package. The oncoPredict R package was employed to predict drug sensitivity based on gene expression levels. This package provides IC50 values for each drug, indicating drug effectiveness based on lower IC50 values.

### 2.8 Construction and validation of prognostic models

A prognostic model was constructed in the TCGA dataset, using LASSO and multiple-factor Cox regression analysis. The coefficients of each gene in the model have been visually represented using a lollipop plot, eliminating the need for separate formulas in the article and improving its aesthetic appeal.

### 2.9 Single-cell analysis

Single-cell analysis was carried out on the Seurat and SCP pipeline (https://github.com/zhanghao-njmu/SCP). Step1: Quality control: Cells with nFeature_RNA < 9000 and percent.mt < 25 were excluded from the analysis. Step2: Batch integration: The harmony R package was used for batch correction and integration of multiple samples. Step3: Annotation: SingleR was employed for the automatic annotation of cell types. Step4: Gene set scoring: The AddModuleScore() function, built-in within the Seurat R package, was used for scoring gene sets.

### 2.10 Cell culture 

We obtained the human breast cancer cell lines, BT-549 and DU4475, from the ATCC. These cells were cultured in Dulbecco's Modified Eagle Medium (DMEM) (Gibco, Grand Island, NY, USA) supplemented with 10% fetal bovine serum (Gibco). The cells were maintained in a 5% CO2 atmosphere at 37 °C. To manipulate the expression of certain genes, we used overexpression plasmids (pcDNA3.1-TMEM31) and si-RNA, which were purchased from GenePharma (Suzhou, China). The transfection was performed using Lipofectamine® 3000 (Invitrogen; Thermo Fisher Scientific) according to the manufacturer's instructions.

### 2.11 Transwell assay

To conduct the Transwell assay, we added an appropriate number of cells in serum-free conditioned medium to the upper chamber. The cells were allowed to incubate at 37°C for 24 hours. Afterward, we removed the cells from the upper chamber and treated the invaded cells with 4% paraformaldehyde. We stained the cells with crystal violet and observed and counted them under a microscope.

### 2.12 Wound healing assay

For the wound healing assay, we seeded the cells in 6-well plates and allowed them to reach a sub-confluent state. After starving the cells in serum-free DMEM for 48 hours, we created a straight wound at the bottom of the plate using a sterile pipette tip. The cells were then cultured in a serum-free medium for 48 hours and observed at 0 and 48 hours using an inverted light microscope.

### 2.13 Cell viability and proliferation assay

To assess cell proliferation, we used a Cell Counting Kit-8 (CCK-8) assay obtained from Shanghai Yeasen Biotechnology Co., Ltd. In 96-well plates, we seeded 2,000 cells and added 10 μl of diluted CCK-8 solution. After incubating the cells with the CCK-8 reagent at 37°C for 1 hour in the dark, we measured the absorbance at 450 nm. Additionally, we detected cell proliferation using the BeyoClickTM EdU Cell proliferation kit, following the manufacturer's instructions from Beyotime Institute of Biotechnology. The cells were stained with Alexa Fluor 488 at room temperature for 30 minutes in the dark and observed using a fluorescence microscope. For cell cycle analysis, cells were fixed with ethanol at 4°C for 48 hours and subsequently stained with PI. Finally, flow cytometry (BD, NJ, USA) was used to evaluate cell cycle.

### 2.14 Statistical analysis

To perform bioinformatics analysis, we utilized R software (version 4.2.3). For statistical analysis of the experimental results, we used Prism 8 (Dotmatics) considering three replications. Two-group comparisons were conducted using unpaired t-tests. A statistically significant difference was defined as a P-value of less than 0.05. The results were presented with error bars indicating the mean ± standard deviation.

## 3 Results

### 3.1 Transcriptional differences of STING pathway-related genes in patients with breast cancer

**Figure 1:**
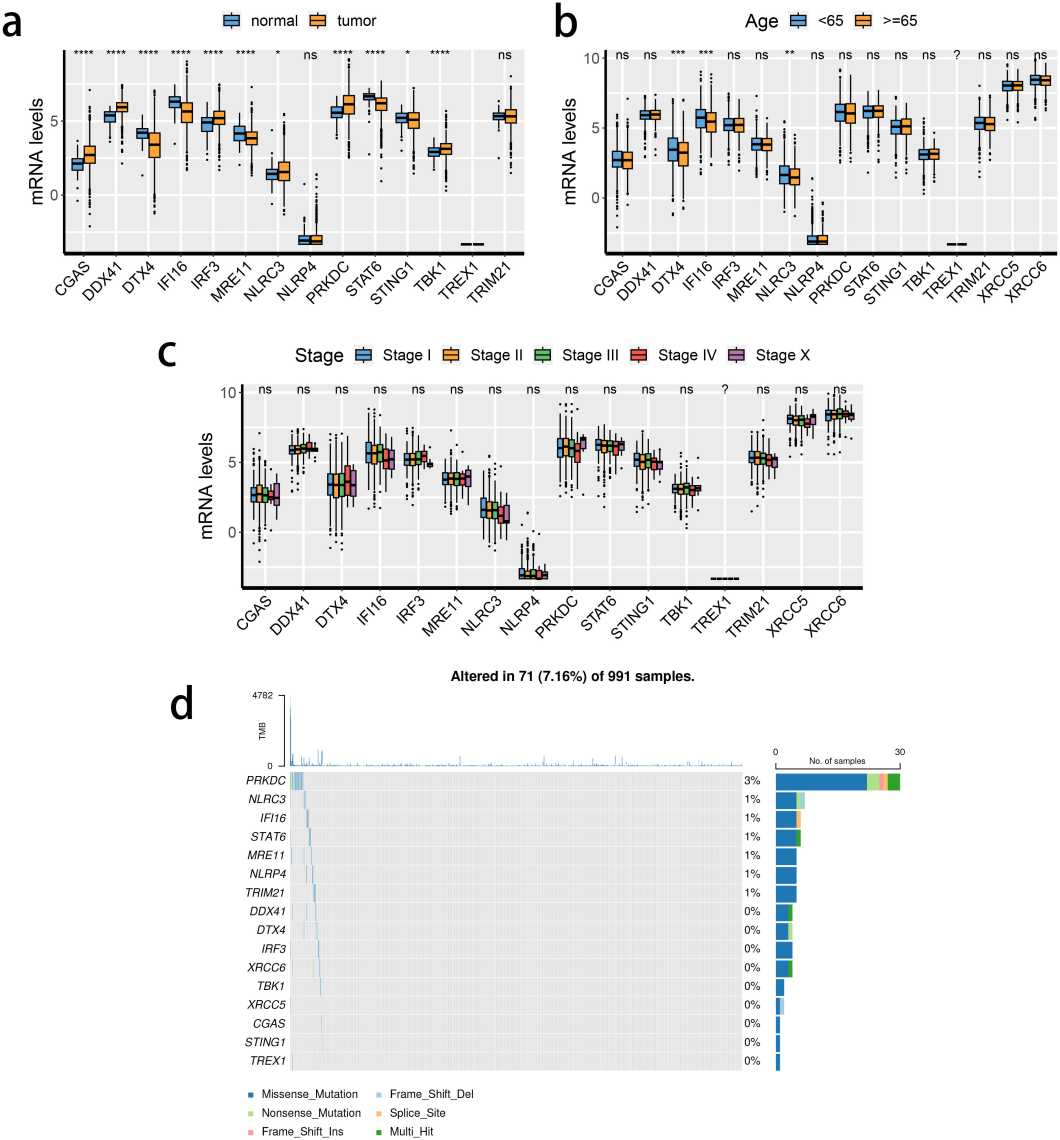
Transcriptional changes of STING pathway-related genes (SRGs) in breast cancer. **(A–C)** The expression patterns of 16 SRGs across tissue types **(A)**, age populations **(B)**, and stages **(C)**. **(D)** Somatic mutation of 16 SRGs.

The transcriptional changes and clinical relevance of SRGs in BRCA patients were examined. Next, the expression patterns of the 16 SRGs across different tissue types (**Figure 1A**), age populations (**Figure 1B**), and clinical stages (**Figure 1C**) in patients with BRCA in the TCGA dataset, and the somatic mutation status of these SRGs (**Figure 1D**). The expression of TREX1 was reduced either in the normal breast or tumorous tissues. Except for NLRP4 and TRIM21, the expressions of the remaining 13 genes between normal and tumorous tissues differed significantly. Of note, the expression of STING1 was down-regulated in the BRCA tissue, while that of TBK1 was enhanced. Moreover, the expression of three SRGs was significantly associated with differences in the age of patients. DDX41, DTX4, and NLRC3 were down-regulated in patients older than 65). However, the relevance of SRGs and clinical staging was not obvious. Among the 16 SRGs, the highest mutation rate was noted in PRKDC in the TCGA cohort.

### 3.2 Consensus clustering analysis of the TCGA-BRCA dataset using STING pathway-related genes

**Figure 2:**
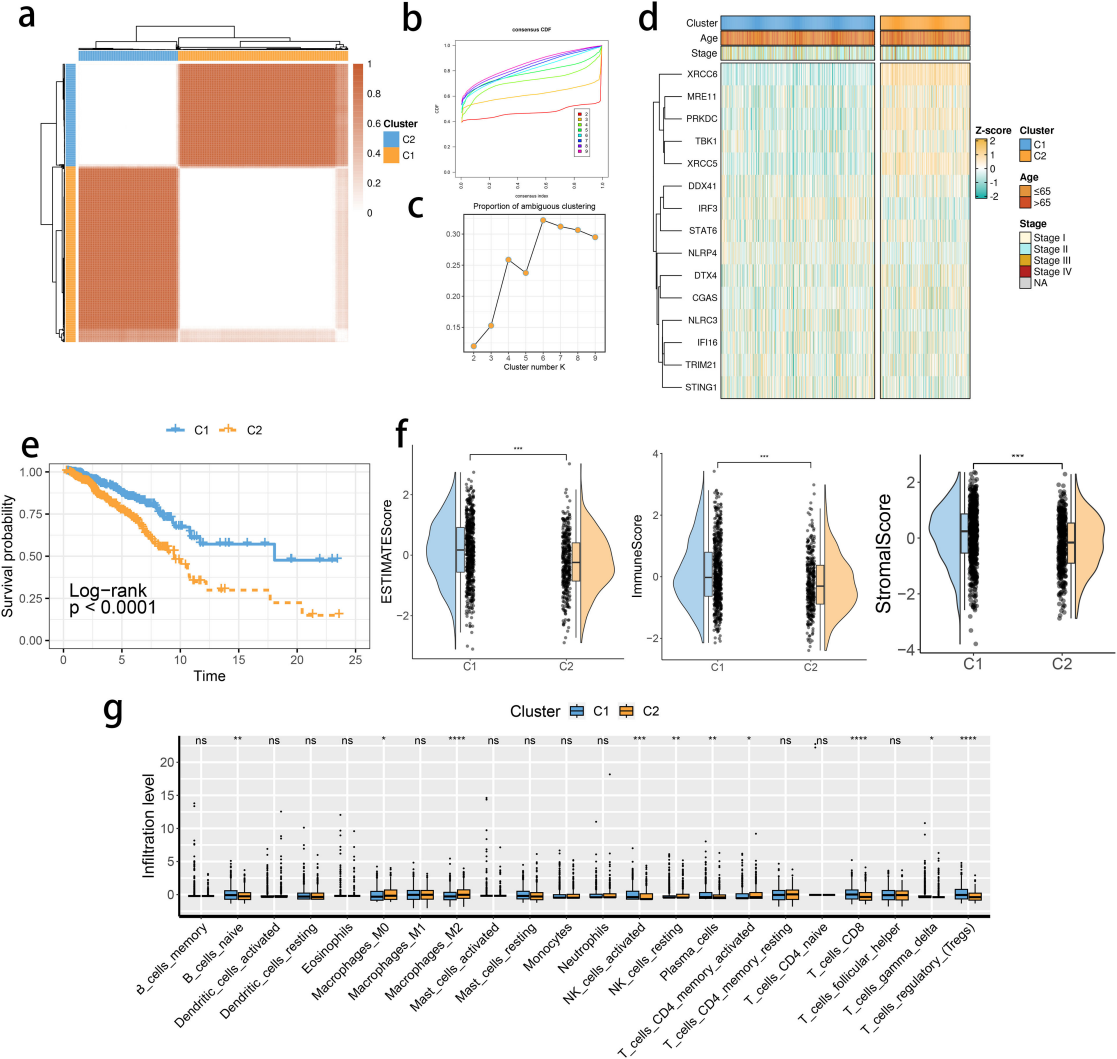
STING pathway-related genes (SRGs)-based molecular clusters with distinct prognosis and tumor microenvironment (TME) landscapes. **(A)** The consensus score matrix of tumor samples in The Cancer Genome Atlas Breast Invasive Carcinoma when the clustering number k = 2. The consensus score represents the intensity of interaction between two samples. **(B, C)** The cumulative distribution function (CDF) curves **(B)** and proportions of ambiguously clustered (PAC) scores **(C)** of the consensus matrix for each k. **(D)** Expression profiles of SRGs and clinicopathological characteristics between clusters. **(E)** Survival analysis between C1 and C2. **(F)** Differences in tumor microenvironment (TME) scores were determined by the ESTIMATE method between clusters. **(G)** Abundance of infiltrating immune cells between clusters. *p< 0.05, **p< 0.01, ***p< 0.001, ****p< 0.0001. ns, not significance.

**Figure 3:**
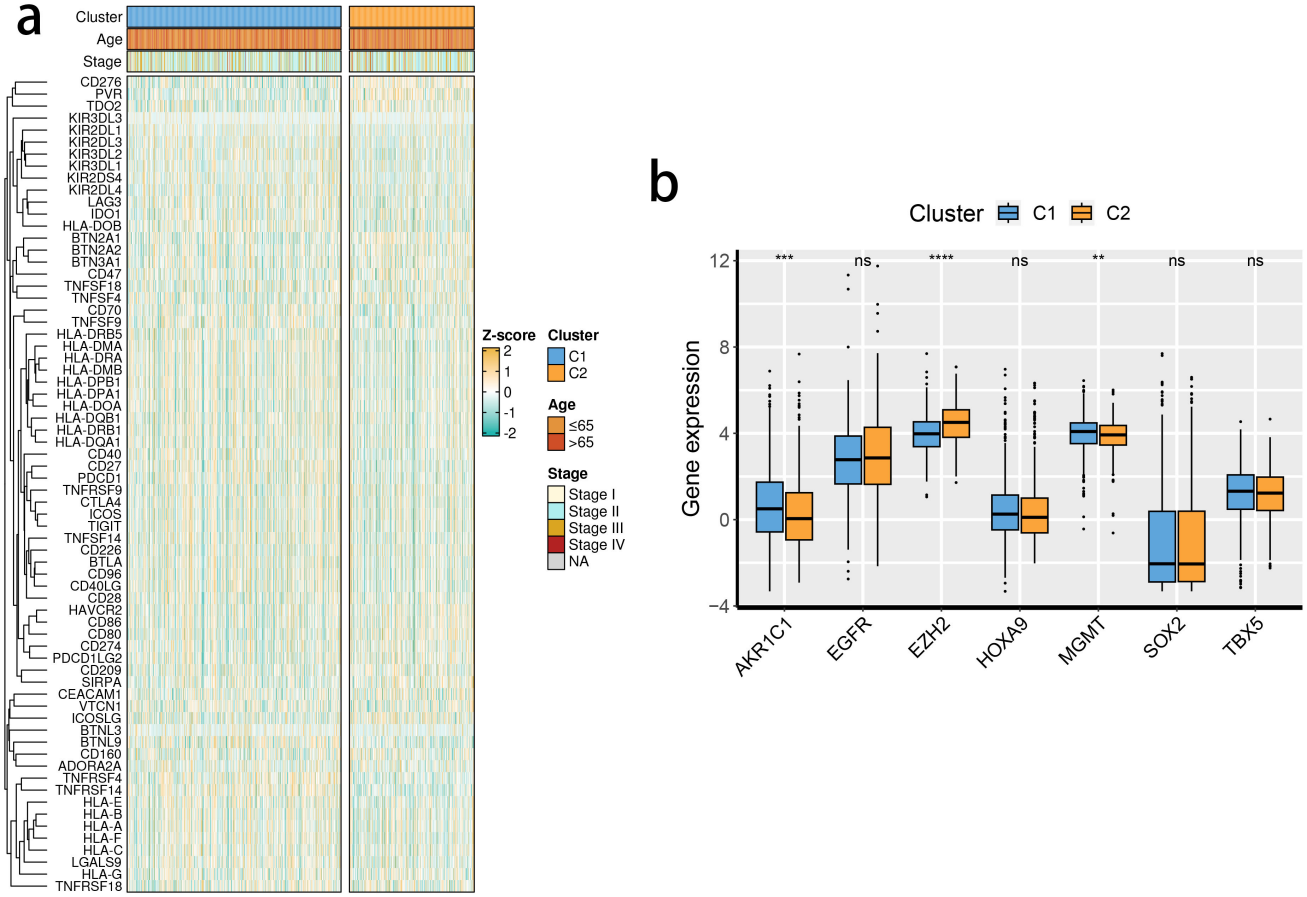
The expression profiles of immune checkpoint inhibitors and chemoradiotherapy sensitivity–related genes between clusters. **(A)** The immune checkpoint inhibitor mRNA expression levels in the 2 identified molecular subclusters. The expression intensity was normalized to the Z score value. **(B)** The chemoradiotherapy sensitivity–related genes mRNA expression levels in the 2 identified molecular subclusters.

The expression levels of the 16 SRGs were used to perform an unsupervised clustering analysis using the ConsensusClusterPlus package to classify the tumor tissues from the TCGA dataset into two distinct groups, C1 and C2 (**Figures 2A–C**). Distinct expression differences in the 16 SRGs between two subclusters were found, highlighting most prominent difference in the XRCC6 expression (**Figure 2D**). We also observed a higher percentage of stage IV and elderly population in the C2 subgroup, which was consistent with survival analysis results and featured a significantly inferior prognosis of C2 (**Figure 2E**). All the three immune-associated evaluations displayed a similar pattern, indicating a more intensively activated TME in the C1 (**Figure 2F**). The immune differences of the two subclusters were further assessed by examining the immune infiltration (**Figure 2G**). M2 macrophages were in significantly higher abundance in the C2 subcluster, while another pivotal immunosuppressive cell type, namely Tregs, was highly abundant in the C1 BRCA tissue. Collectively, these results suggested M2 macrophages may potentially have a predominant role in shaping immunosuppressive TME in BRCA, rather than Tregs. A potential association between STING pathway activation and M2 infiltration is also suggested. We assessed the differences in the expression of the immune checkpoint inhibitors. The expression of CD276, PVR, and TDO2 were prominently up-regulated in C2 (**Figure 3A**), while AKR1C1 and MGMT were highly expressed in the C2 subgroup (**Figure 3B**), suggesting a potential difference in radiotherapy sensitivity in these two subgroups.

**Figure 4:**
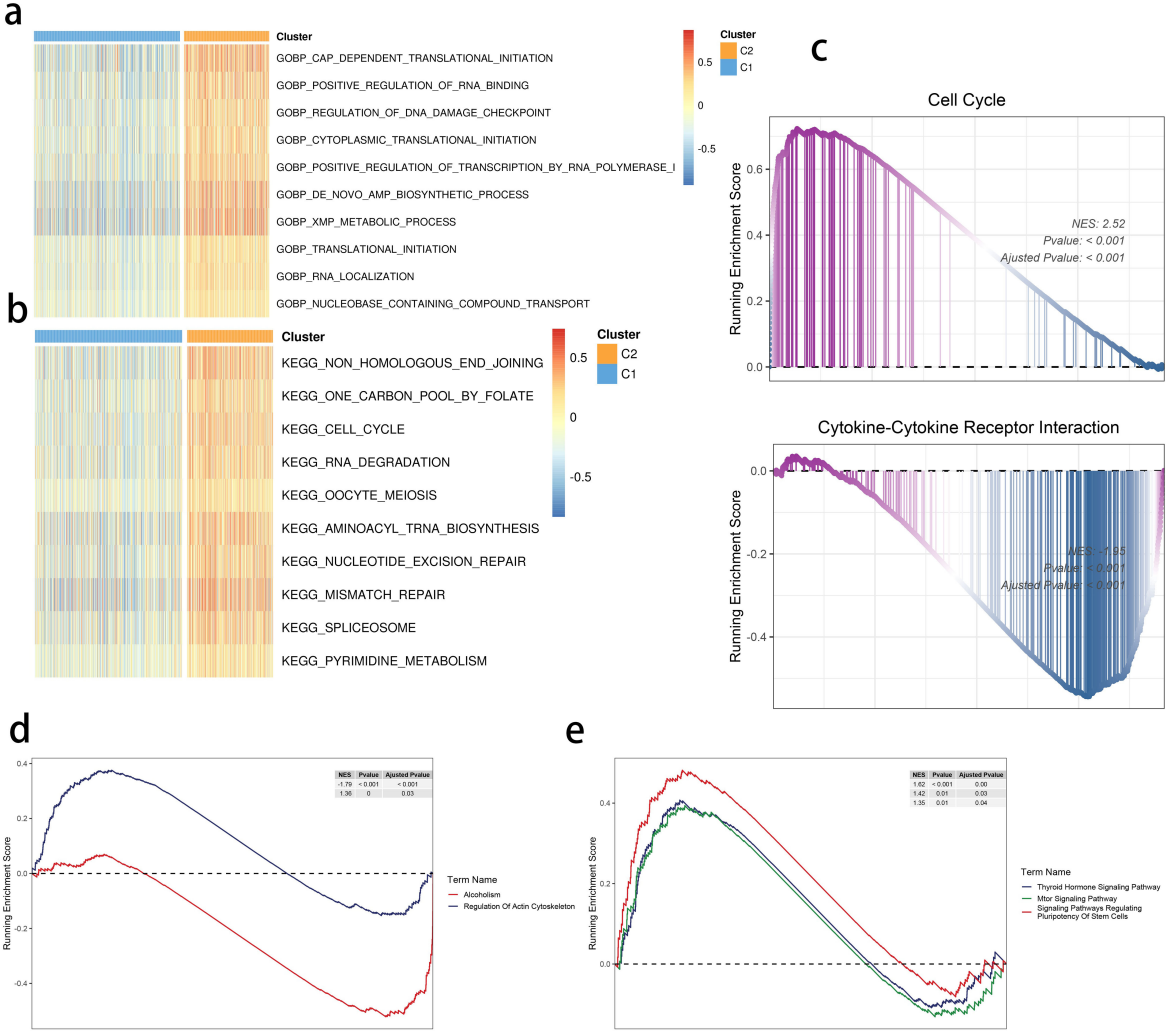
SRGs-based molecular clusters with dysregulated pathways and biological process. **(A, B)** GSVA of GO-BP **(A)** and KEGG (b) terms between 2 identified molecular subclusters. **(C)** GSEA of significant hallmarks enriched in SRG C2. **(D, E)** GSEA of significant dysregulated pathways enriched in SRG C2.

We found translational initiation and highly activated *de novo* AMP biosynthetic process within the C2 subgroup (**Figure 4A**). Moreover, a similar pattern of the cell cycle and RNA degradation was noted in the C2 group (**Figure 4B**). We focused on these functional differences and performed the Gene Set Enrichment Analysis, highlighting a consistent result (**Figure 4C**). Moreover, the mammalian target of rapamycin signaling, cell stemness, and regulation of actin were also differentially regulated in the two clusters (**Figures 4D, E**).

### 3.3 Detection of gene clusters related to the STING pathway

**Figure 5:**
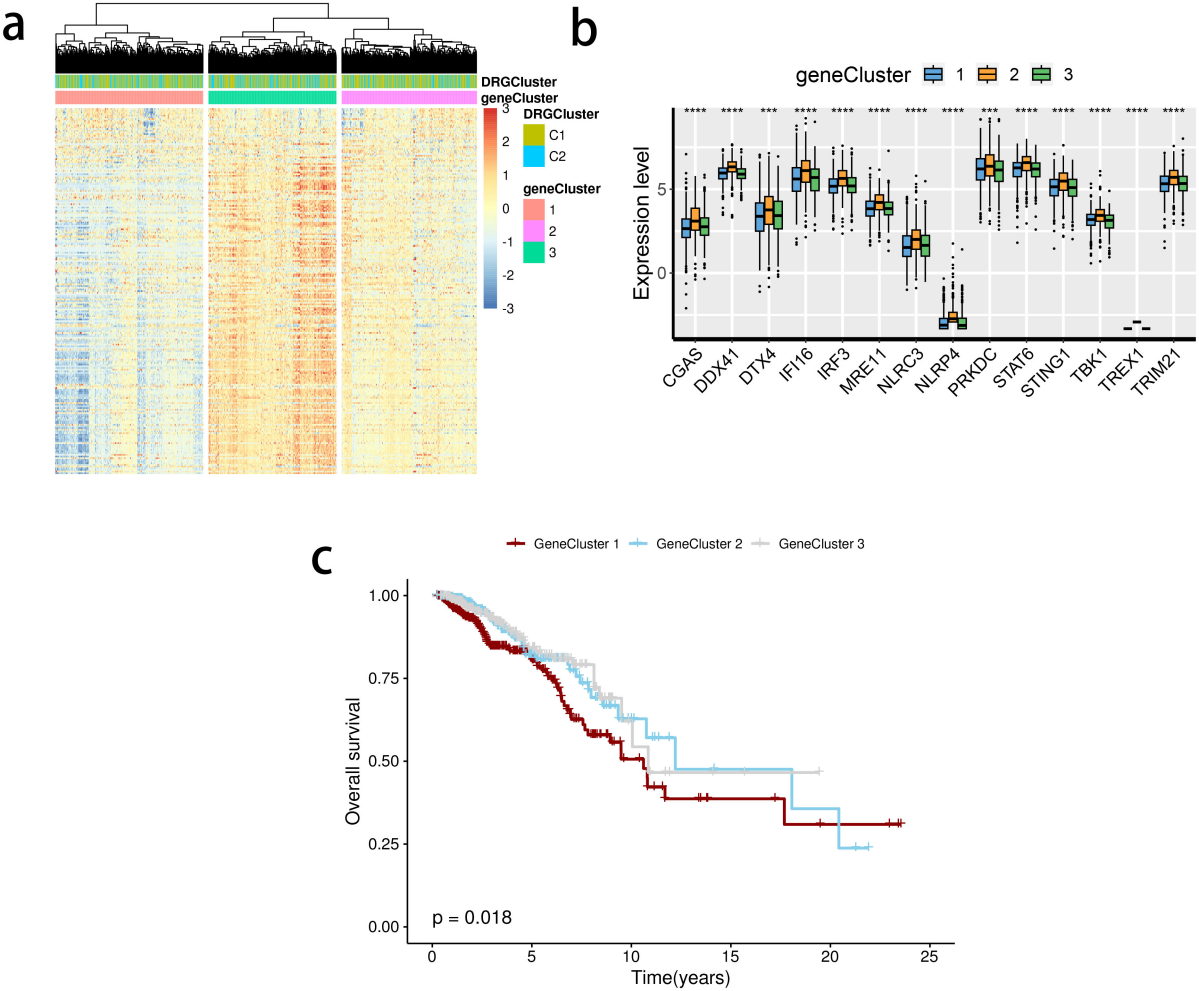
Detection of gene clusters related to the stimulator of interferon genes (STING) pathway. **(A)** The expression profiles of DEGs and the distribution of SRG clusters among gene clusters 1 to 3. **(B)** mRNA levels of 16 STING pathway-related genes between groups. **(C)** Survival analysis among three gene clusters. ns, not significance, **p< 0.01, ***p< 0.001, ****p< 0.0001.

The limma package was used to detect differentially expressed genes (DEGs) between the C2 and C1 groups (**Figure 5A**), and a total of three subclusters were identified. The expression of the mentioned SRGs was markedly different between these groups (**Figure 5B**). Gene cluster 1 had the most inferior survival outcome (**Figure 5C**).

### 3.4 Development and validation of the STING pathway-related prognostic model

**Figure 6:**
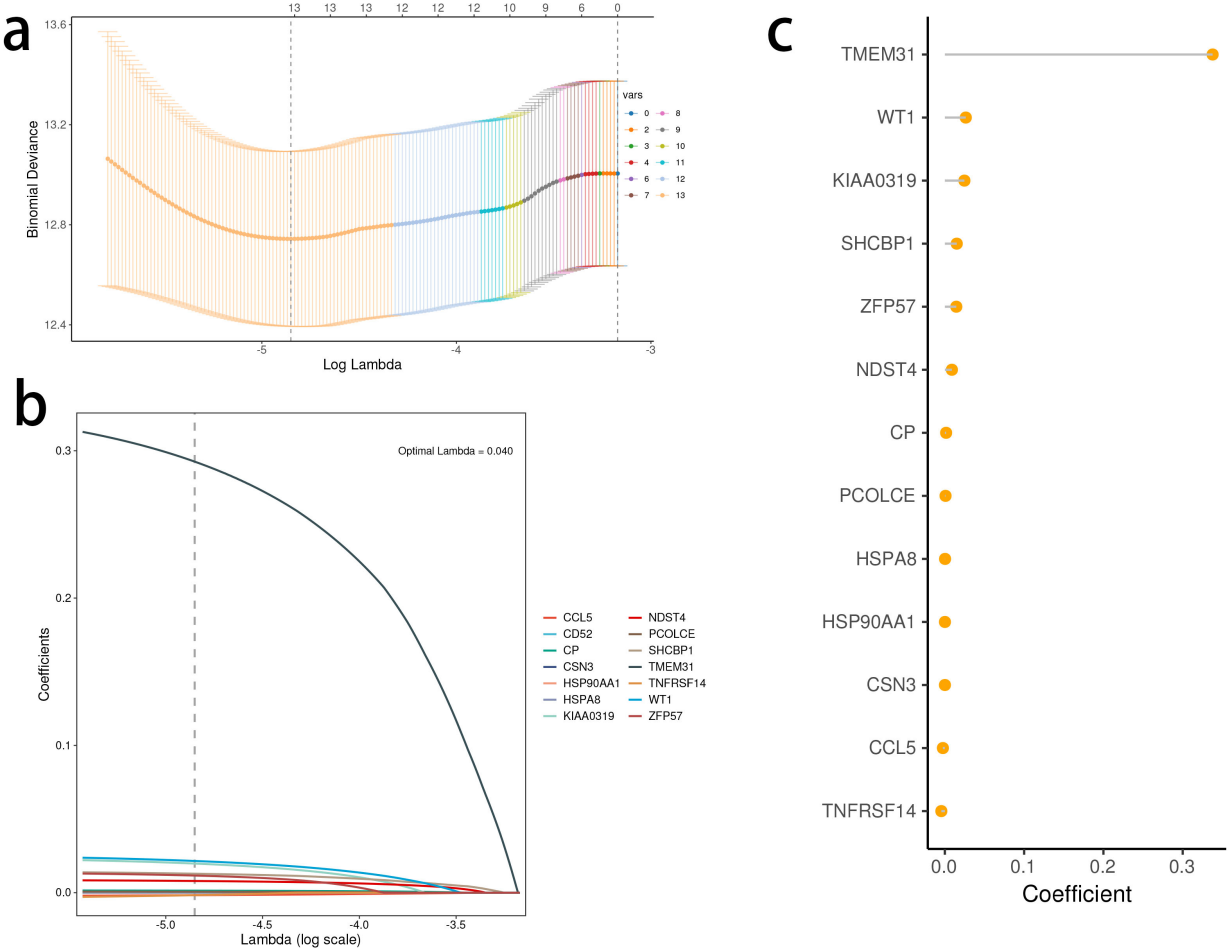
Development of the stimulator of interferon genes (STING) pathway-related prognostic model. **(A, B)** The selection of prognostic hub genes based on the optimal parameter λ that was obtained in the least absolute shrinkage and selection operator regression analysis. **(C)** Lollipop chart of the coefficients of signature genes determined by the multiCox regression analysis.

**Figure 7:**
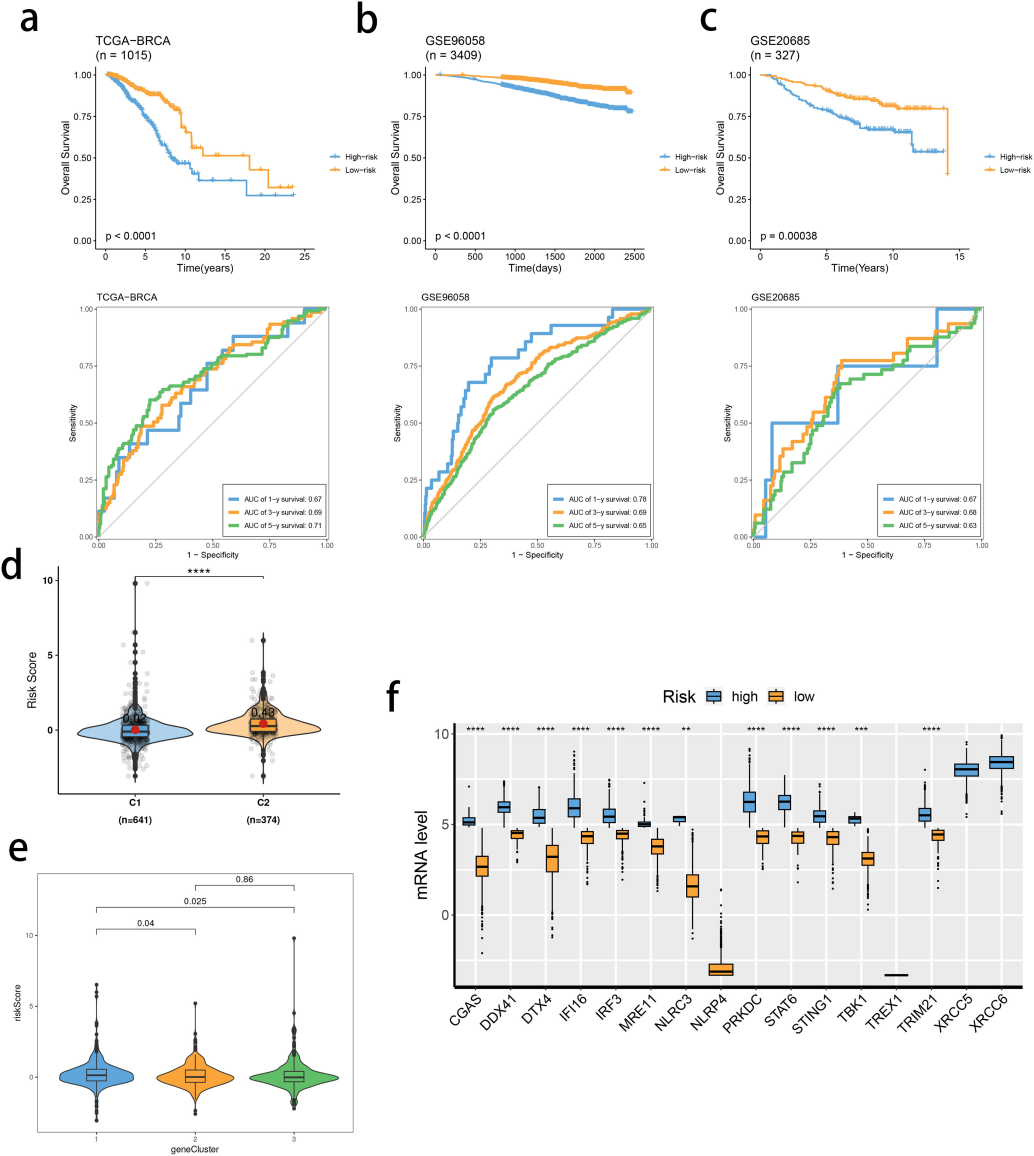
Validation of the stimulator of interferon genes (STING) pathway-related prognostic model. **(A–C)** Survival analysis and prognostic performance of the model in the three cohorts. **(D, E)** Distribution of risk scores between SRG clusters **(D)** and gene clusters **(E)**. **(F)** mRNA levels of 16 SRGs between risk groups. ns, not significance, **p< 0.01, ***p< 0.001, ****p< 0.0001.

Based on the identified DEGs related to the STING pathway, we developed a prognostic model using LASSO and multiple-factor regression analyses (**Figures 6A–C**). The highest coefficient value was noted for TMEM31, followed by those of WT1 and KIAA0319. The prognostic model displayed high prognosis stratification potency in the training set (TCGA dataset, **Figure 7A**). The prognostic model was validated using two external GEO datasets (**Figures 7B, C**), highlighting an area under the ROC curve value > 0.7 at multiple time points. Consistent with our expectation, the C2 subgroup had a higher risk score value (**Figure 7D**). Subgroup 1 also was characterized by a higher value of risk score, in accordance with the inferior clinical outcome (**Figure 7E**). We determined the expression of 16 SRGs in the BRCA groups of different risk values. The high-risk group exhibited notable up-regulation of STING1 and TBK1 (**Figure 7F**).

### 3.5 Correlation analysis investigating tumor microenvironment from the perspective of the risk score value

**Figure 8:**
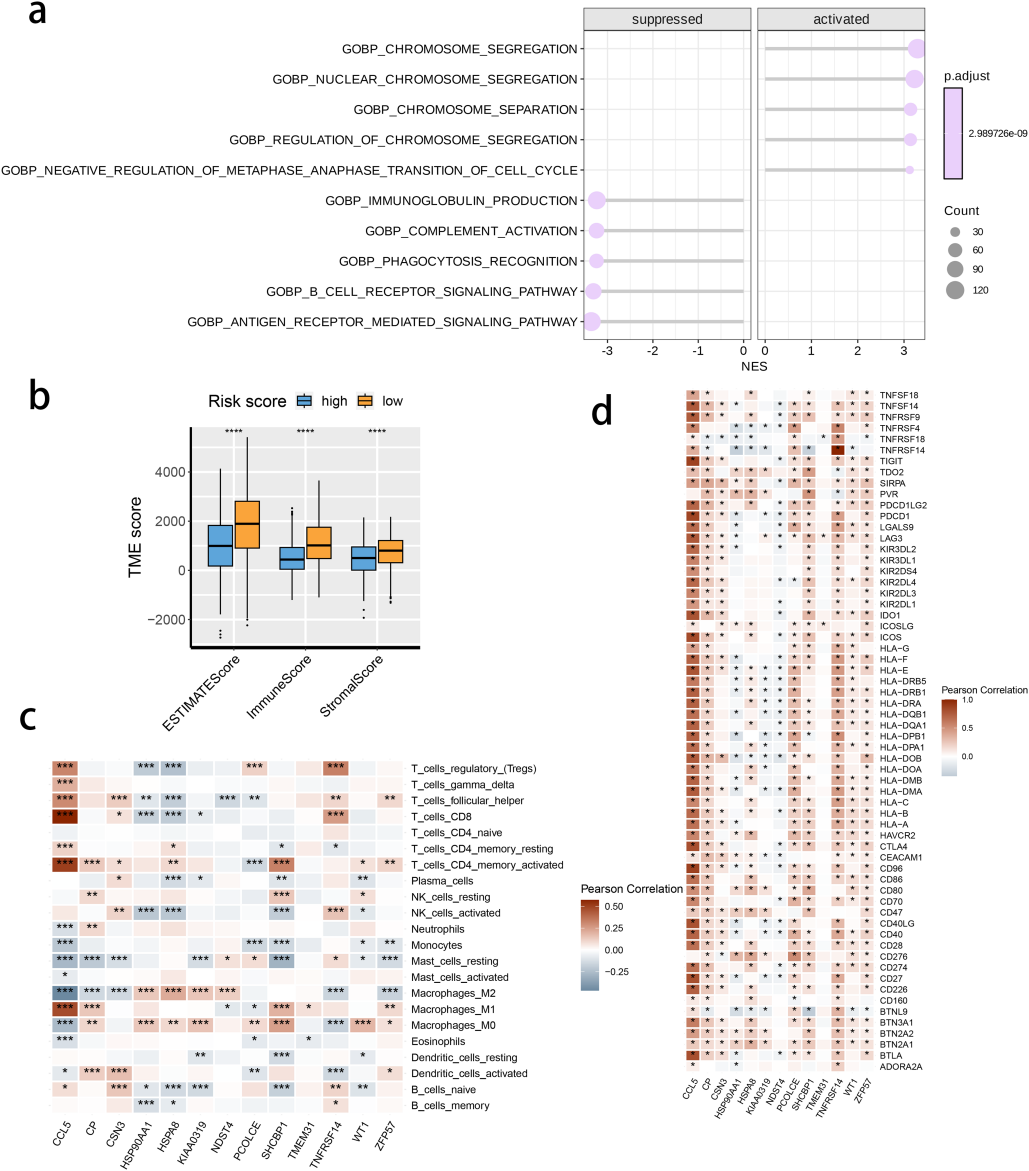
The correlation between the risk score and tumor microenvironment score. **(A)** The activated or suppressed hallmarks in the STING pathway-related genes (SRG) C2. **(B)** Differences in TME scores were determined by the ESTIMATE method between the two risk groups. **(C)** Correlations between the risk score and the abundance of immune cells. **(D)** Correlations between the risk score and the immune checkpoint genes.

**Figure 9:**
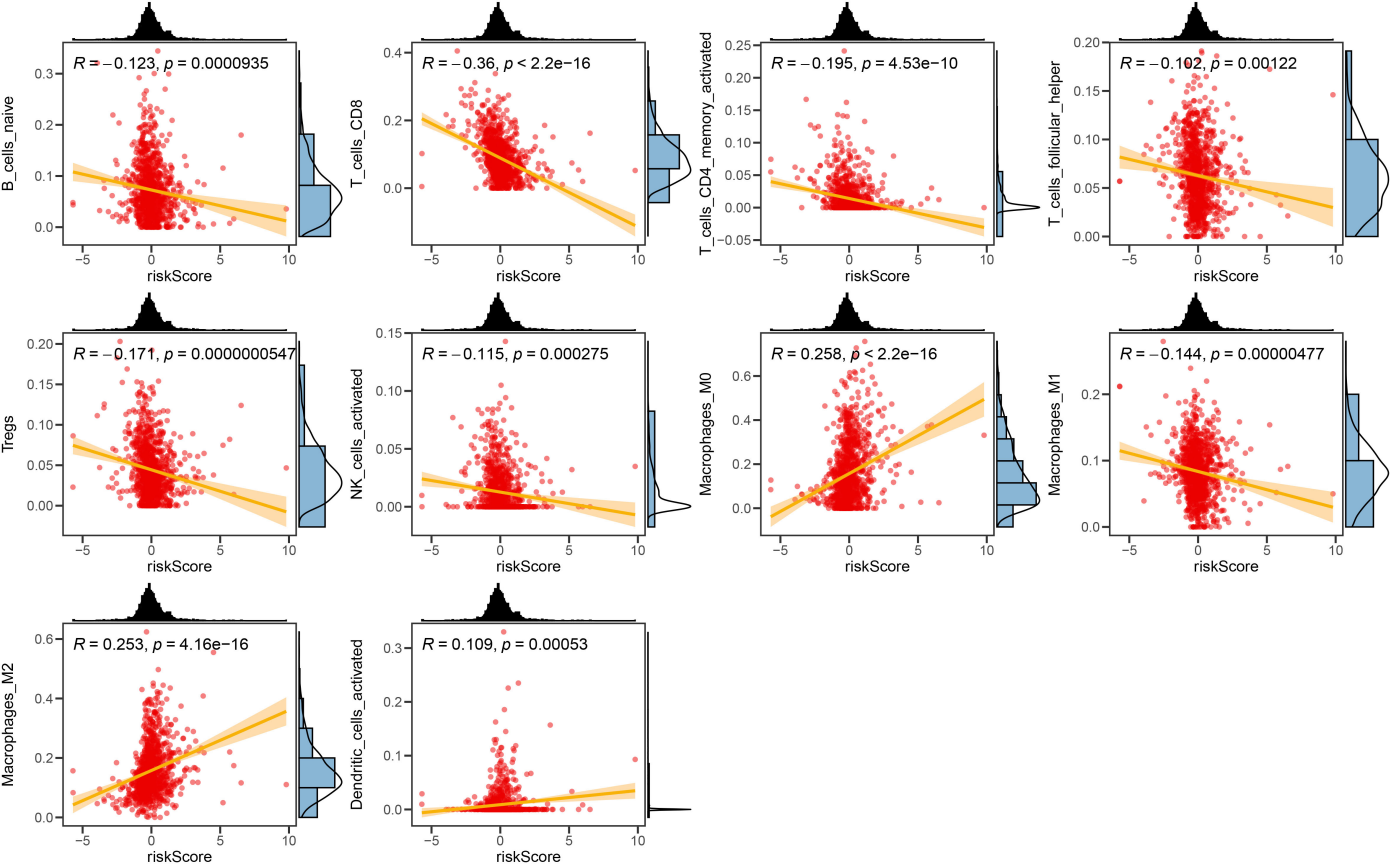
The correlation between the Risk score and immune cell sets. The scatter plots displaying the correlative relationship between the abundance of different infiltrated immune cells and risk score value.

**Figure 10:**
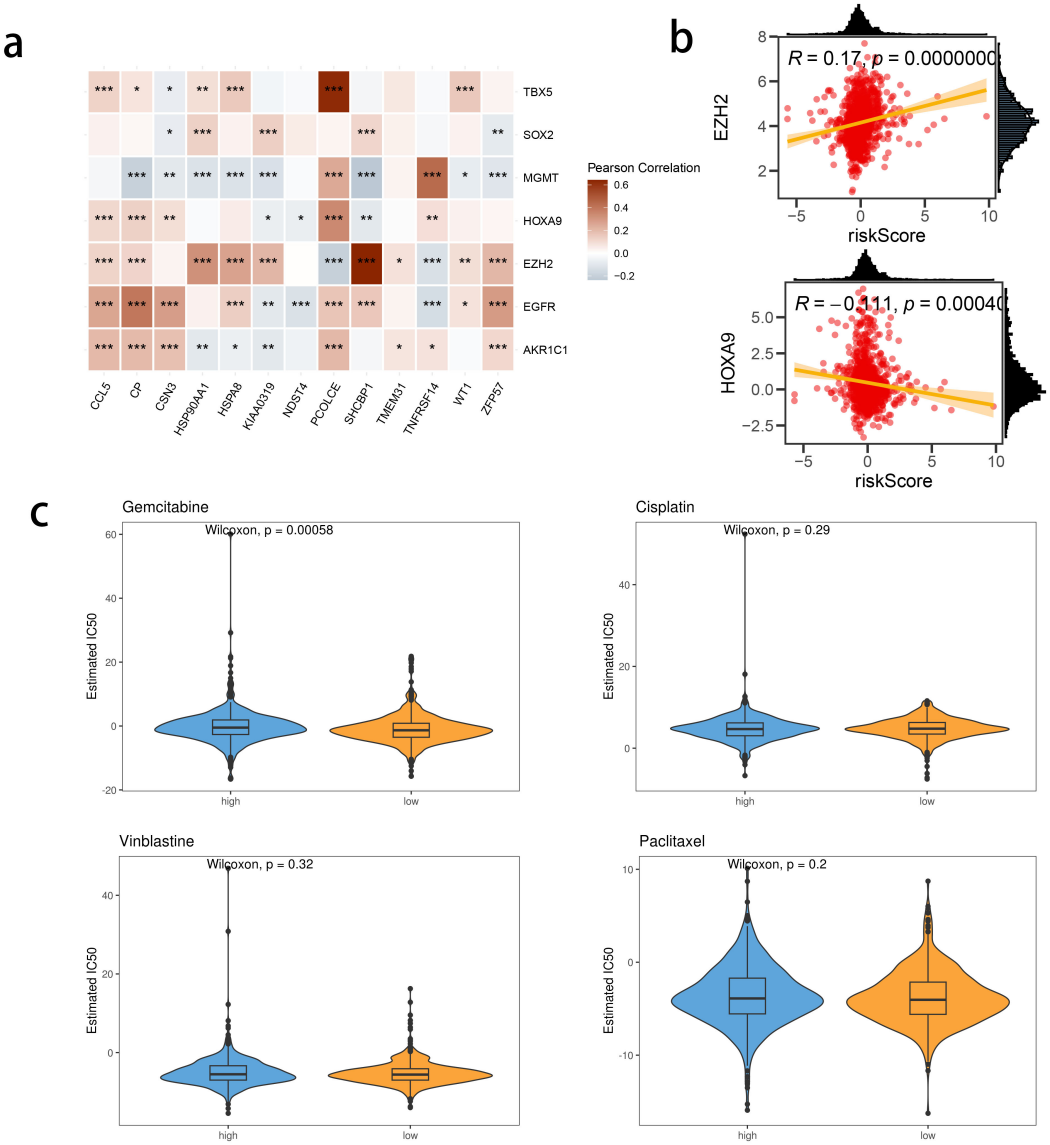
The correlation between risk score and chemotherapy sensitivity-related genes. **(A)** The correlations between prognostic model genes and chemotherapy sensitivity-related genes. **(B)** The correlations between the genes related to chemotherapy sensitivity and the Risk score. **(C)** Predicted IC50 between risk groups.

We investigated the correlation between TME and risk score value to delineate the underlying mechanism of the strong prognosis stratification potency of our model. We found that higher risk score significantly suppressed the complement activation, immunoglobulin production, and phagocytosis, suggesting a significantly hampered immune activation in the patients with BRCA having higher risk scores (**Figure 8A**). All three immune evaluations consistently showed a similar result (**Figure 8B**). The expression of CCL5, CP, ZFP57, and TNFRSF14 were highly correlated with M1 macrophage infiltration, while negatively associated with M2 abundance (**Figure 8C**). Among the hub genes, TMEM31, featuring the highest coefficient value, was positively correlated with the expression of LAG3 and ICOSLG, both immunosuppressive marker genes (**Figure 8D**). The infiltration of CD8, CD4, and B cells was distinctly decreased in the higher risk score, while M2 was positively correlated with the risk score (**Figure 9**). Among the hub genes, SHCBP1 had a high association with EZH2 expression, while PCOLCE was highly associated with TBX5 (**Figure 10A**), suggesting that these genes could act as potential novel biomarkers to assess the sensitivity to BRCA chemotherapy. Moreover, EZH2 exhibited a high correlation with risk value (**Figure 10B**). The high-risk group had a higher IC50 value for gemcitabine, suggesting a more favorable outcome in the low-risk group (**Figure 10C**).

### 3.6 A highly activated STING pathway-related signature in scRNA-seq datasets of breast cancer

**Figure 11:**
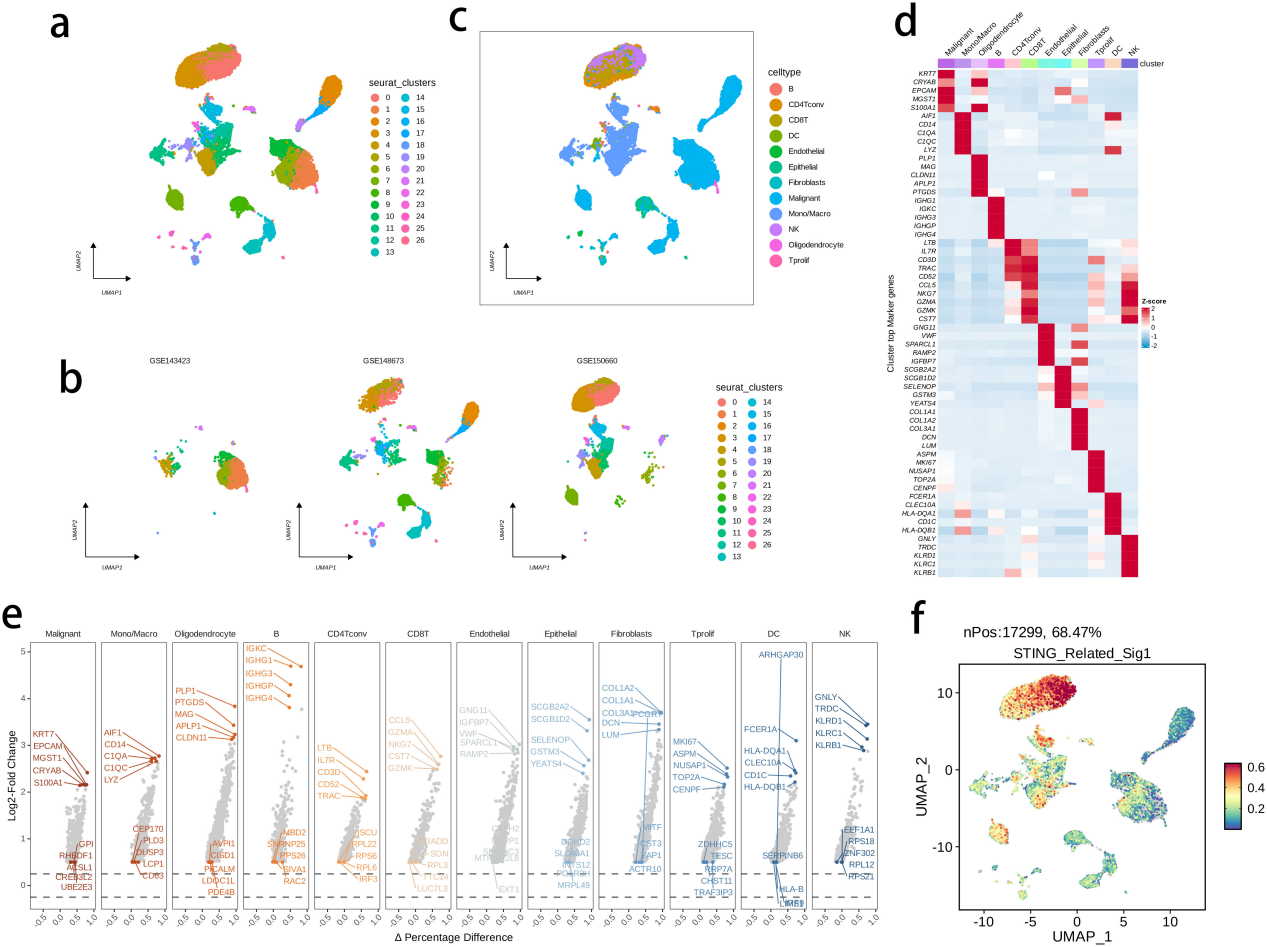
The highly activated STING pathway-related signature in single-cell RNA (scRNA)-seq datasets of BRCA. **(A, B)** UMAP visualization of cells from three public BRCA scRNA-seq cohorts. **(C)** 12 major cell types were annotated. **(D)** Heatmap showing the top five markers of each major cell set. **(E)** Volcano plots illustrating the downregulated and upregulated genes of each major cell set. **(D)** The expression of signature genes at single cell level was determined by the AddModuleScore() function in Seurat.

The activity of the prognostic model was further investigated at the single-cell level, by analyzing three public single-cell RNA-seq datasets. We performed quality control, dimensionality reduction, and clustering analysis, and generated a total of 27 subclusters (**Figures 11A, B**), that were subsequently annotated into 12 main cell types (**Figure 11C**) in terms of the expression levels of marker genes as displayed in **Figures 11D, E**. The intratumor NK cells exhibited a highly activated STING pathway-related signature in the CD8 T cells, further supporting the clinical relevance of the prognostic model with BRCA TME (**Figure 11F**). -

### 3.7 A versatile role of TMEM31 in regulating BRCA proliferation and migration

**Figure 12:**
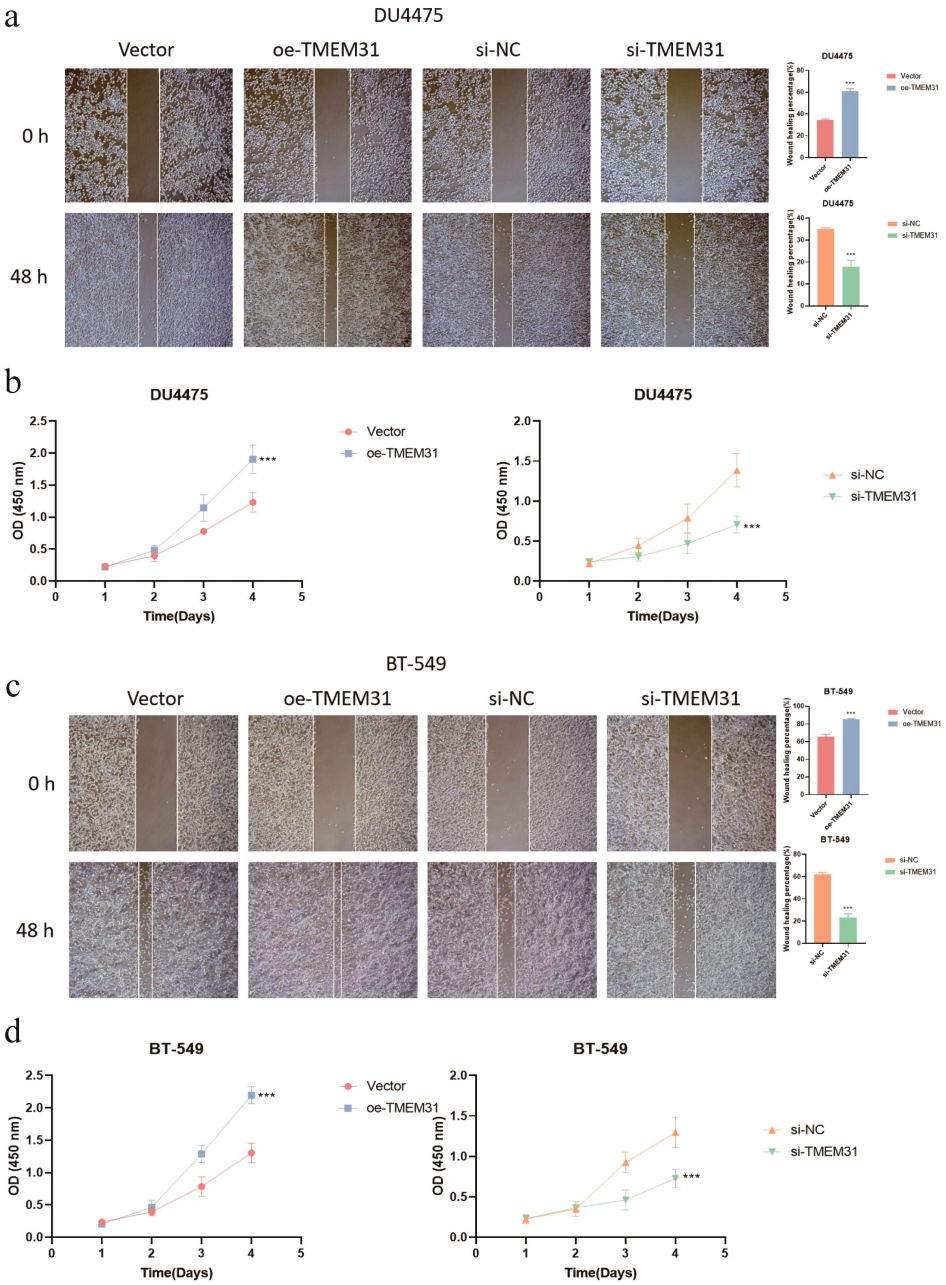
A versatile role of TMEM31 in regulating BRCA proliferation and migration. **(A)** The wound healing assay investigating whether the up-regulation and down-regulation of TMEM31 affected the migratory capability of the DU4475. **(B)** CCK8 analysis assessing the cell proliferation rate of DU4475 with up-regulation and down-regulation of TMEM31. **(C)** The wound healing assay investigating whether the up-regulation and down-regulation of TMEM31 affected the migratory capability of the BT-549. **(D)** The CCK8 analysis assessing whether the up-regulation and down-regulation of TMEM31 affected the proliferation rate of BT-549. (*p< 0.05, **p< 0.01, ***p< 0.001, ns, not significance.).

**Figure 13:**
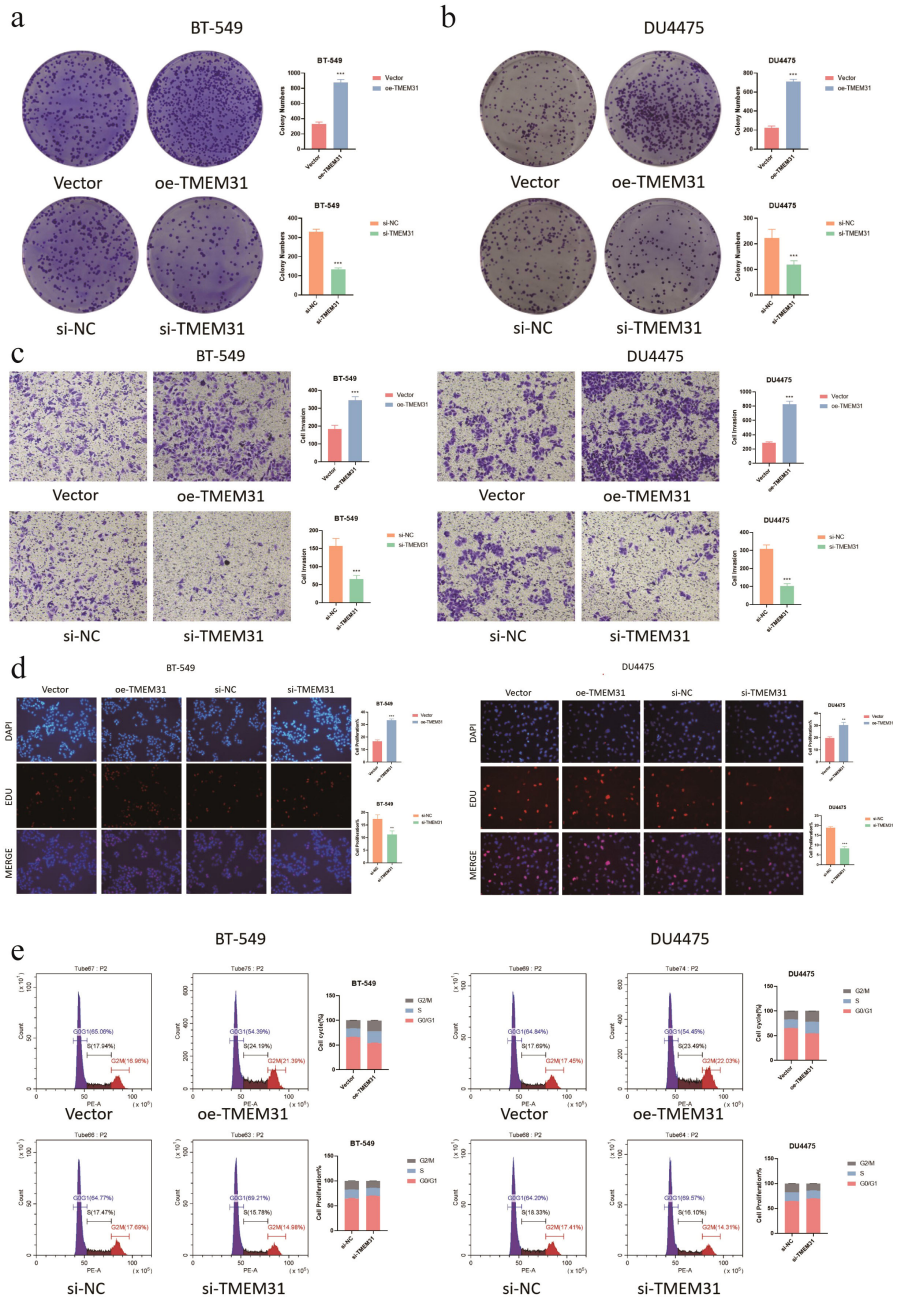
Further validation of TMEM31 in regulating BRCA proliferation and migration. **(A, B)** CFA analysis investigating whether the loss and overexpression of TMEM31 contributed to alteration in the colony formation capability of BT-549 (a) and DU4475 **(B)**. **(C)** Transwell analysis assessing the transmembrane migration of BT-549 and DU4475 with up-regulation and down-regulation of TMEM31, respectively. **(D, E)** The EDU assay investigating whether the up-regulation and down-regulation of TMEM31 affected the proliferation of the BT-549 and DU4475. **(D)** The flow cytometry assessing whether the up-regulation and down-regulation of TMEM31 affected the cell cycle progression of BT-549 and DU4475. (*p< 0.05, **p< 0.01, ***p< 0.001, ns, not significance.).

In this study, we identified TMEM31 as the main risk factor in our prognosis model, hence we investigated the role of TMEM31 in regulating the proliferation and migration of BRCA cells. To examine the role of TMEM31 in mediating the migratory capability of DU4475 cells, we performed a wound healing assay, where we observed up-regulation of TMEM31 resulted in the enhanced migratory capability (**Figure 12A**). Additionally, up-regulation and down-regulation of TMEM31 prompted and hampered the cell proliferation rate of DU4475, respectively (**Figure 12B**). Similarly, we conducted a wound healing assay and CCK8 analysis to explore the effects of up-regulation and down-regulation of TMEM31 on the migratory capability and proliferation rate of BT-549 cells, revealing similar results (**Figures 12C, D**). Furthermore, we further validated the role of TMEM31 in BRCA cells by conducting a colony formation assay. Both BT-549 and DU4475 cells were analyzed and showed that the loss or overexpression of TMEM31 affected their colony formation capability (**Figures 13A, B**). We further conducted the Transwell analysis to investigate the tridimensional migration regulated by TMEM31. In both the BT-549 and DU4475 cells, manipulating the levels of TMEM31 contributed to altered the transmembrane migration (**Figure 13C**). Additionally, we used the EDU assay to investigate the effects of TMEM31 on the proliferation (**Figure 13D**). We employed flow cytometry to evaluate whether the up-regulation and down-regulation of TMEM31 influenced the cell cycle progression of BT-549 and DU4475 cells (**Figure 13E**). We observed a higher proportion of G2/M cells in the BT-549 and DU4475 cells. Overall, our findings demonstrate the versatile role of TMEM31 in regulating the proliferation and migration of BRCA cells. These results provide valuable insights into the potential therapeutic targeting of TMEM31 in BRCA treatment.

## 4 Discussion

BRCA treatment strategies are evolving, with a focus on targeting STING activation triggered by DNA damage. One of the promising approaches for BRCA treatment involves targeting the nuclear receptor NR1D1 (REV-ERBα) to enhance antitumor immune responses. One study reported that deletion of Nr1d1 in mouse models resulted in increased tumor growth and metastasis, primarily driven by Nr1d1 loss in tumor cells. NR1D1 promotes cytosolic DNA fragment accumulation and activates cGAS-STING signaling, leading to increased production of type I interferons (IFNs) and immune chemokines (8). Ectonucleotide pyrophosphatase/phosphodiesterase 1 (ENPP1) drives BRCA growth and metastasis by suppressing the anti-tumoral immunity mediated by extracellular cGAMP-STING signaling. Inhibition of ENPP1 function slows tumor growth, prevents metastasis, and selectively blocks the cGAMP hydrolysis function of ENPP1, replicating the effects of complete ENPP1 knockout (9). JMJD8, an endoplasmic reticulum protein, inhibits the STING-mediated immune response in BRCA. JMJD8 competes with TBK1 for binding to STING, and prevents the formation of the STING-TBK1 complex, leading to reduced expression of type I IFN1 and IFN-stimulated genes. This immune evasion mechanism promotes breast tumorigenesis (10). Overexpression of DNA repair proteins in triple-negative BRCA can affect the efficacy of chemotherapy and sensitivity to DNA repair inhibitors (11). For example, IFI16 has shown promise in inducing a STING-mediated immune response in triple-negative breast cancer (BRCA). However, the cGAS-STING pathway induced by DNA repair activation is correlated with poor patient survival (12). These findings indicate a bidirectional role of the cGAS-STING pathway in BRCA, emphasizing the need to understand its regulation. One of the promising approaches for cancer therapy is the inhibition of DNA repair and promotion of DNA damage response (DDR) progression to activate the cGAS-STING pathway. PARP1 inhibitors and other DDR-targeting drugs have demonstrated efficacy in treating breast and ovarian cancers. Paclitaxel, a commonly used BRCA treatment, activates the cGAS-STING pathway through its effects on cell division. These findings highlight the potential of targeting the cGAS-STING pathway in BRCA cells.

In addition to its critical role in BRCA cancer cells, STING activation was also highly active in the TME. Elevated levels of tumor-infiltrating lymphocytes within the TME play a crucial role in treating BRCA (13). Differential analysis of tumor compartments in patients with triple-negative BRCA responsive to chemotherapy exhibit high levels of STING protein, indicating its presence in BRCA TME and its potential as a treatment target (14). Under the condition that the cGAS-STING pathway is activated within the BRCA TME, increased persistence of T helper/IL-17-producing CD8+ T -generated CAR-T cells is observed in the TME and enhanced tumor control (15). These reports support our observation that a highly active STING pathway-related signature was found in the CD8 T cells and intratumor NK cells at single-cell resolution. The intra-tumoral DCs produce IFN-β upon induction by the STING protein, which initiates and recruits T cells into the TME. Consequently, STING agonists hold significant promise for reshaping the immunosuppressive TME by reversing its immunosuppressive nature and sensitizing BRCA to immunotherapy.

Rather than Tregs, M2 macrophages might have a predominant role in shaping immunosuppressive TME in BRCA, (16). CHI3L1, secreted by M2 macrophages, promotes the metastasis of gastric and BRCA cells. CHI3L1 interacts with the interleukin-13 receptor α2 chain on the membrane of cancer cells, activating the mitogen-activated protein kinase signaling pathway. This leads to increased enhanced of matrix metalloproteinase genes, promoting tumor metastasis. CHI3L1 levels were also significantly higher in the serum of patients with gastric cancer and BRCA compared to healthy donors (17). The tumor-adipose microenvironment (TAME) is a novel microecosystem characterized by lipid metabolism dysfunction. The infiltration of M2-like macrophages in TAME is linked to poor survival in BRCA. Fatty acid transporters in TAME predict BRCA survival and are associated with macrophage function. Further, we identified lipid-associated macrophages (LAMs) in TAME expressing lipid metabolism genes and markers through scRNA sequencing and spatial transcriptomics. LAMs display an M2-like macrophage signature, lipid accumulation, and enhanced phagocytosis. Depleting LAMs in allograft cancer mouse models synergizes with anti-PD1 therapy. This defines a unique macrophage subtype in TAME with distinct clinical outcomes (18). Glycyrrhetinic acid (GA), a compound found in licorice, exhibits potential anti-cancer effects. In BRCA, GA inhibits the M2-like polarization of TAMs without affecting M1-like polarization. GA further reduces the expression of M2 markers and pro-angiogenic molecules in M2 macrophages, while promoting c-Jun N-terminal kinase 1/2 signaling. GA also suppresses tumor growth, angiogenesis, and lung metastasis in mice (19). Thus, these findings suggest that targeting M2 polarization could be an effective strategy in treating BRCA.

In our study, a higher IC50 value of gemcitabine in the high-risk group suggests a more favorable outcome in the low-risk group. Gemcitabine, a pyrimidine antimetabolite, has shown activity in metastatic BRCA singly as well as in combination regimens (20). Phase II trials have demonstrated the activity of gemcitabine in pretreated and unpretreated patients. In a Phase III trial, gemcitabine was inferior to epirubicin in elderly patients (21); however, when combined with taxanes like paclitaxel or docetaxel, gemcitabine showed high activity, improving response rate, the time to disease progression, quality of life, and survival (22). Currently, in a phase II trial, trilaciclib administered before gemcitabine plus carboplatin (GCb) improved overall survival in patients with metastatic TNBC. The ongoing phase III PRESERVE 2 trial is evaluating the efficacy and safety of trilaciclib when administered before GCb in patients with locally advanced unresectable or metastatic TNBC (23). This approach will potentially offer a new treatment strategy for patients with TNBC and potentially improve outcomes. In the case of advanced BRCA, gemcitabine monotherapy has not been reported to significantly improve patient survival. Singh et al. developed a combination regimen of gemcitabine with imiquimod and delivered using hyaluronic acid-based nanoparticles, to stimulate immune cells for anticancer activity. The combination showed enhanced anticancer effects *in vitro* as well as in a mouse model of BRCA. The findings suggest that imiquimod can enhance the efficacy of gemcitabine through the activation of immune cells to suppress tumors (24).

## 5 Conclusion

We developed and validated a STING pathway-related prognostic model that accurately predicts BRCA patient outcomes. These findings have important implications for the personalized treatment and management of BRCA patients.

## Data Availability

The datasets presented in this study can be found in online repositories. The names of the repository/repositories and accession number(s) can be found in the article/supplementary material.
